# Y-LineageTracker: a high-throughput analysis framework for Y-chromosomal next-generation sequencing data

**DOI:** 10.1186/s12859-021-04057-z

**Published:** 2021-03-09

**Authors:** Hao Chen, Yan Lu, Dongsheng Lu, Shuhua Xu

**Affiliations:** 1 Key Laboratory of Computational Biology, Shanghai Institute of Nutrition and Health, University of Chinese Academy of Sciences, Chinese Academy of Sciences, Shanghai, 200031 China; 2 School of Life Sciences, Fudan University, Shanghai, 200433 China; 3 School of Life Science and Technology, ShanghaiTech University, Shanghai, 201210 China; 4 Center for Excellence in Animal Evolution and Genetics, Chinese Academy of Sciences, Kunming, 650223 China; 5 Henan Institute of Medical and Pharmaceutical Sciences, Zhengzhou University, Zhengzhou, 450052 China; 6 Collaborative Innovation Center of Genetics and Development, Fudan University, Shanghai, 200438 China

**Keywords:** Y-chromosome DNA, NGS, NRY haplogroup, Y-STR, Population genetics

## Abstract

**Background:**

Y-chromosome DNA (Y-DNA) has been used for tracing paternal lineages and offers a clear path from an individual to a known, or likely, direct paternal ancestor. The advance of next-generation sequencing (NGS) technologies increasingly improves the resolution of the non-recombining region of the Y-chromosome (NRY). However, a lack of suitable computer tools prevents the use of NGS data from the Y-DNA studies.

**Results:**

We developed Y-LineageTracker, a high-throughput analysis framework that not only utilizes state-of-the-art methodologies to automatically determine NRY haplogroups and identify microsatellite variants of Y-chromosome on a fine scale, but also optimizes comprehensive Y-DNA analysis methods for NGS data. Notably, Y-LineageTracker integrates the NRY haplogroup and Y-STR analysis modules with recognized strategies to robustly suggest an interpretation for paternal genetics and evolution. NRY haplogroup module mainly covers haplogroup classification, clustering analysis, phylogeny construction, and divergence time estimation of NRY haplogroups, and Y-STR module mainly includes Y-STR genotyping, statistical calculation, network analysis, and estimation of time to the most recent common ancestor (TMRCA) based on Y-STR haplotypes. Performance comparison indicated that Y-LineageTracker outperformed existing Y-DNA analysis tools for the high performance and satisfactory visualization effect.

**Conclusions:**

Y-LineageTracker is an open-source and user-friendly command-line tool that provide multiple functions to efficiently analyze Y-DNA from NGS data at both Y-SNP and Y-STR level. Additionally, Y-LineageTracker supports various formats of input data and produces high-quality figures suitable for publication. Y-LineageTracker is coded with Python3 and supports Windows, Linux, and macOS platforms, and can be installed manually or via the Python Package Index (PyPI). The source code, examples, and manual of Y-LineageTracker are freely available at https://www.picb.ac.cn/PGG/resource.php or CodeOcean (https://codeocean.com/capsule/7424381/tree).

**Supplementary Information:**

The online version contains supplementary material available at 10.1186/s12859-021-04057-z.

## Introduction

The human Y-chromosome plays a crucial role in understanding human evolution and genetics [1]. The NRY is one of the most informative regions of the human genome, making it an effective instrument for the study of paternal inheritance [2]. The single-nucleotide polymorphisms (SNPs) and short tandem repeats (STRs) on the NRY have been used as significant markers to trace direct paternal ancestral lineages and reflect the peculiarities of historical male behaviors [3]. In particular, with the increasing popularity of NGS platforms in recent years, the NRY has provided researchers increasingly informative markers to track human paternal lineages [4].

The basic analysis for human paternal lineage study is to infer NRY haplogroups, which were mainly defined by a set of specific Y-SNPs. On the one hand, with the accumulation of Y-chromosome NGS data, several tools have been developed to meet such a need: AMY-tree [5], clean-tree [6], Yleaf [7], and HaploGrouper [8]. These tools support the function of NRY haplogroup inference, but the lack of subsequent genetic analysis based on NRY haplogroup results prevents further tracing and understanding of the patrilineality. On the other hand, with the continuous updating of Y-SNP markers and the topology of the human Y-chromosome tree, such as ISOGG Y-DNA tree (https://www.isogg.org/tree) and YFull tree (https://www.yfull.com/tree), NRY haplogroups classified by some tools were rendered outdated by the latest version. Indeed, it is essential to keep updating the Y-SNP markers and NRY haplogroup nominations up to date for paternal lineage studies. Y-STR polymorphisms provide more informative haplotypes than Y-SNPs do, and they have been widely used in forensics and population genetics [3, 9, 10]. However, at present, the acquisition of Y-STR data relies mainly on experimentation based on polymerase chain reaction (PCR) amplification, and most of the current analyses do not utilize Y-STRs from the high-throughput Y-chromosome sequencing data [11]. With the advance of NGS technologies, various tools have been developed for genotyping STRs from NGS data in recent years. These tools were designed mainly for clinically applications such as TRhist [12], STR-FM [13] and Dante [14], forensic applications such as STRait Razor [15, 16], or trait-association studies such as lobSTR [17], HipSTR [18] and popSTR [19, 20]. Under these circumstances, however, there is a lack of comparable efforts for population genetics and evolutionary studies. In particular, a specific tool or pipeline is required to efficiently make full use of Y-chromosome sequencing data and provide comprehensive analyses with the combination of Y-SNPs and Y-STRs to infer paternal lineage and history in human populations.

To facilitate the Y-DNA data analyses in the context of evolutionary and medical studies, we developed Y-LineageTracker, an open-source and user-friendly tool that standardizes the Y-DNA analysis workflow. Y-LineageTracker assembles a collection of functions applied for human Y-DNA analyses (Tables 1 and 2) and supports multiple inputs and output formats that can communicate with commonly used tools for human genetic analyses such as PAML [21], MEGA [22], and Network (https://www.fluxus-engineering.com), providing a framework to fully analyze human Y-chromosome sequencing data from both Y-SNP and Y-STR levels. The results of the evaluation indicated that Y-LineageTracker can perform faster and more comprehensively with higher resolution and better compatibility than other methods.

**Table 1:** Functions implemented in Y-LineageTracker

Function	Command	Description
NRY haplogroup classification	Classify	Classify NRY haplogroups from BAM or VCF file
Clustering analysis	Cluster	Perform clustering analysis for NRY haplogroups
Phylogeny analysis	Phylo	Perform phylogeny analysis for NRY haplogroups
Y-STR genotyping	Genostr	Genotype Y-STRs from BAM or VCF indels
Network analysis	Net	Perform network analysis for Y-STR haplotypes
Statistical analysis	Stat	Perform statistical analysis for Y-STR haplotypes
Time estimation	Time	Estimate NRY haplogroup divergence time
	Tmrca	Estimate TMRCA of Y-STR haplotypes

Y-DNA analysis functions implemented in Y-LineageTracker. Each function is corresponding to a command

**Table 2:** Statistical methods applied in Y-LineageTracker

Function	Method or Statistics	Reference
Clustering Analysis	Principal component analysis (PCA)	[23]
	Multidimensional scaling (MDS)	[24, 25]
Phylogeny Analysis	Maximum parsimony (MP)	[26, 27]
	Identity by state (IBS)	[28, 29]
Network Analysis	Median-joining (MJ)	[30]
Statistical Analysis	Haplotype diversity (HD)	[31]
	Mean pairwise distance (MPD)	[32]
	Fixation index (Fst)	[33]
	Genetic diversity (Gst)	[34, 35]
	Analysis of molecular variance (AMOVA)	[36]
	Haplogroup prediction from Y-STRs	[37]
Time Estimation	Mcmctree in PAML	[21]
	Rho statistics	[38]
	The average squared difference (ASD)	[39, 40]

The published Y-DNA analysis methods applied in Y-LineageTracker

## Implementation

**Fig. 1:**
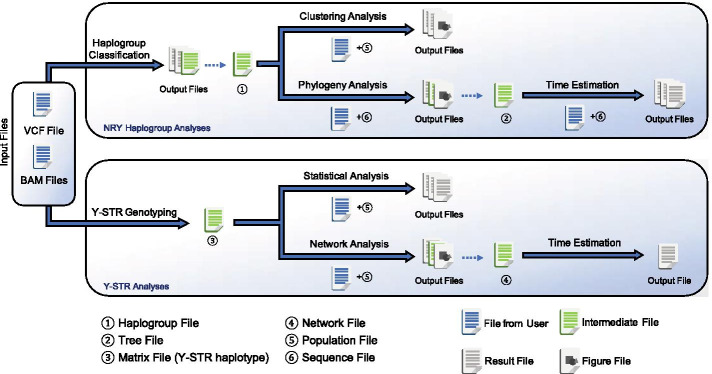
Workflow and main functions of Y-LineageTracker. The Y-LineageTracker supports taking VCF or BAM files as input data to perform NRY haplogroup classification and Y-STR genotyping. The outputs of haplogroup classification can be used as input data for subsequent clustering and phylogeny analysis, and the outputs of phylogeny analysis can be further used for time estimation. Similarly, the outputs of Y-STR genotyping can be used for statistical analysis and network analysis, and the output of network analysis can be further used for TMRCA estimation

Y-LineageTracker is a command-line tool implemented in Python3 with popular NGS data processing and scientific computational Python packages. Y-LineageTracker makes use of Y-chromosome sequencing data and enables subsequent analyses for NRY haplogroups and Y-STRs. As shown in the workflow and main functions of Y-LineageTracker (Fig. 1), Y-LineageTracker contains two analysis modules: NRY haplogroup module and Y-STR module. As for NRY haplogroup module, Y-LineageTracker takes VCF or BAM files as input data for NRY haplogroup classification. The non-sequencing genotype data in VCF are also supported. Y-LineageTracker matches all possible mutations based on the ISOGG Y-DNA tree (2019 version) and determines an optimal track from the terminal haplogroup to Y-Adam, the ancestral Y-chromosome haplotype without any mutation. After NRY haplogroup classification is complete, Y-LineageTracker further 1) applies principal component analysis (PCA) or multidimensional scaling (MDS) clustering method at a specific haplogroup level, 2) constructs a rooted bifurcating phylogenetic tree, and 3) estimates divergence time of haplogroups. For Y-STR module, Y-LineageTracker provides a function to genotype Y-STRs from VCF indels or BAM files; multiple Y-STR genotyping panels are supported, including commonly used commercial Y-STR genotyping kits (Table 3 and Additional file 1). Because comparing genetic differences among populations is a general method of directly unraveling population history, common population statistical analyses for Y-STR haplotypes are included in Y-LineageTracker, such as haplotype diversity, mean pairwise distance, different types of genetic distance, and analysis of molecular variance (AMOVA). Y-LineageTracker also provides a Y-STR haplotype reference data set to infer the most likely NRY haplogroup using the Bayesian approach [37]. Also, users can perform median-joining network analysis and generate a haplotype network plot or a fdi file, which can be directly used as input to draw a network plot or estimate time in the Network software (https://www.fluxus-engineering.com).

**Table 3:** Y-STR genotyping panels supported in Y-LineageTracker

Panel	Number of Loci	Description
Minimal	9	DYS19, DYS385a, DYS385b, DYS389I, DYS389II, DYS390, DYS391, DYS392, DYS393
PowerPlex Y	12	Minimal + DYS437, DYS438, DYS439
Yfiler	17	PowerPlex Y + DYS448, DYS456, DYS458, DYS635 YGATAH4
PowerPlex Y23	23	Yfiler + DYS481, DYS533, DYS549, DYS570, DYS576, DYS643
Named	92	All the possible Named Y-STR loci on the Y-chromosome
All	2, 451	All the possible Y-STR loci on the Y-chromosome

Multiple Y-STR genotyping panels are supported in Y-LineageTracker and can be used for Y-STR genotyping. The Y-STR genotyping panels refers to YHRD (https://yhrd.org) [41], STRBase (https://strbase.nist.gov) [42] and lobSTR (http://lobstr.teamerlich.org) [17]

### NRY haplogroup classification with tracking lineage

Before starting NRY haplogroup classification, we firstly divide markers of each haplogroup on the ISOGG Y-DNA tree into two classes: key and ordinary. The key markers are those traditionally used or tested in labs, which are displayed in bold on the ISOGG Y-DNA tree.

To classify haplogroup accurately, Y-LineageTracker first collects all the haplogroups of which most markers are derived alleles. Then, the program selects haplogroups with key mutation marker as key haplogroups and trace possible lineages from the Y-Adam to different downstream and determine the confident terminal haplogroup as the classification result for each sample. When tracing lineages, the program has two criteria: 1) calculating the matching rate of each possible downstream haplogroup and select those are confident; 2) calculating the tracking rate based on the resolution of terminal haplogroup and the number of matched haplogroups in this track. Consider $t$ is the tracking rate for each lineage track:

$$t = \frac{{\mathop \sum \nolimits_{i = 1}^{r} R_{i} }}{r}$$

$$R = \left\{ {\begin{array}{*{20}l} 1 \hfill & {\quad \left( {if\,\frac{n}{m} \ge U} \right)} \hfill \\ 0 \hfill & {\quad \left( {if\,\frac{n}{m} < U} \right)} \hfill \\ \end{array} } \right.$$

where $r$ is the resolution of the terminal haplogroup in each track, $n$ is the number of sites matched to mutations of the haplogroup, $m$ is the number of all the possible mutations for this haplogroup, and $U$ is a cutoff value, the matching rate of haplogroups greater than this value will be selected. As a result, haplogroups with a relatively higher matching rate will be traced, while haplogroups with a lower matching rate but matched to derived allele due to mismatches or sequencing error will be excluded. Next, consider the optimal lineage track that includes haplogroups from the terminal one to the main trunk as $T_{f}$:

$$T_{f} = T_{{max\left( {t_{1} ,t_{2} , \ldots ,t_{s} } \right)}}$$

where $s$ represents the number of possible tracks, thus track with the most confident tracking rate will be determined. As a result, the final lineage track is a coherent chain with confident haplogroups and a maximum tracking rate.

Finally, the program can also try to detect potential final terminal haplogroup compared to the current terminal key one from its downstream haplogroups without key mutation markers. Samples whose final terminal is not equal to key haplogroup will be annotated in the mutation column of the output file.

For NGS data in VCF, the program also considers the alleles of which all samples are the same to the reference genome and thus not shown in the VCF file. If there are no key haplogroups matched due to the input data is non-sequencing data, or the resolution is relatively low, the program can also apply a similar approach to matched ordinary haplogroups and perform classification analysis. The details of the haplogroup simplification method is provided in Additional file 2. Besides, we also provide the option *–ref*, which allows users to customize the NRY marker panel in classification module, although we attempt to keep updating NRY haplogroup maker panel following the latest version of the ISOGG Y-DNA tree.

### Clustering analysis based on NRY haplogroups

Y-LineageTracker provides clustering analysis to show population structure at the population level. The program takes haplogroup file and population file as input files, then calculates the haplogroup frequency of each population. Based on the haplogroup frequency data, the program calculates eigenvectors and eigenvalues using the PCA method or calculates population pairwise F_ST_ then computes positions in the embedding space using the MDS method. For example, to identify population structure using PCA, we can start clustering analysis by a very simple command: *LineageTracker cluster –hg file.hg -p file.pop –method pca*. After the running is finished, the program outputs the results of the calculation and a scatter plot to show the relationship among populations.

Because the resolution of NRY haplogroups may be different among samples, users can also specify the level of haplogroup resolution using *–level* option to simplify haplogroups at the relatively same resolution by different simplification methods. For example, command *LineageTracker cluster –hg file.hg -p file.pop –method pca –level 3* simplifies haplogroups at level 3. More details of simplification methods in clustering analysis are provided in Additional file 2.

### Phylogeny construction based on NRY haplogroups

Y-LineageTracker provides a function to construct a phylogenetic tree from NRY haplogroups. To construct an NRY phylogenetic tree more confidently and quickly, we here propose a two-step strategy: First, Y-LineageTracker constructs a preliminary tree using haplogroup classification results as a priori inputs, and then it modifies it with multiple sequence alignment data to produce a confident bifurcating tree. The program reads the input NRY haplogroups and converts input haplogroup data to tree structure by assigning each haplogroup to the corresponding position of the ISOGG Y-DNA tree (2019 version). Next, the redundant nodes of the tree will be pruned and modified. However, some nodes of the tree may be polytomies (nodes with more than two children) because some samples have the same haplogroup. Thus, we recommended users to input sequence alignment data as well to construct a bifurcating tree. For example, command *LineageTracker phylo –hg file.hg –seq file.fasta –seq-format fasta* uses the information of NRY haplogroup and sequence to accurately construct a bifurcating NRY phylogenetic tree. Y-LineageTracker applies tree construction methods such as the unweighted pair group method with arithmetic mean (UPGMA), maximum parsimony and identity-by-state, to generate bifurcating sub-trees in post order (from leaves to root). A sub-tree is a portion of the tree data, the updated sub-tree will be used to replace the polytomy while keeping the original tree structure unchanged. Users can change the tree construction method by the *–method* option. For example, command *LineageTracker phylo –hg file.hg –seq file.fasta –seq-format fasta –method mp* applies a method of maximum parsimony to construct the NRY phylogenetic tree.

### Estimation for haplogroup divergence time

PAML mcmctree [21] is wrapped in Y-LineageTracker to apply the Bayesian MCMC algorithm for estimating haplogroup divergence time. To execute PAML mcmctree program, Y-LineageTracker firstly generates a control file from arguments and then takes required input files (a sequence alignment file, a tree file, and a control file) to perform Bayesian estimation. The tree file should be a rooted bifurcating tree without branch length in newick format. To run PAML mcmctree, fossil calibration is also required. Y-LineageTracker provides commonly used calibration time of haplogroups of Y-DNA tree main trunks to automatically use built-in calibration information based on the input tree. The outputs of time estimation include raw outputs of the mcmctree and results summarized from them, which gives the estimated time of each tree node.

### Y-STR genotyping

To genotype Y-STR loci from Y-chromosome sequencing data, users just need to provide input file and the version of reference genome as arguments and type a very simple command: *LineageTracker genostr –bam file.bam -b 38*. Y-LineageTracker also provides a different genotyping panel for Y-STR genotyping (Table 3). The Y-STR genotyping panel of hg19 refers to YHRD [41], STRBase [42] and lobSTR [17], then we apply LiftOver software (https://genome.sph.umich.edu/wiki/LiftOver) to get reference panel of hg38 as well. Unnamed Y-STRs with repeat numbers less than four or irregular motif arrangements were dropped.

**Fig. 2:**
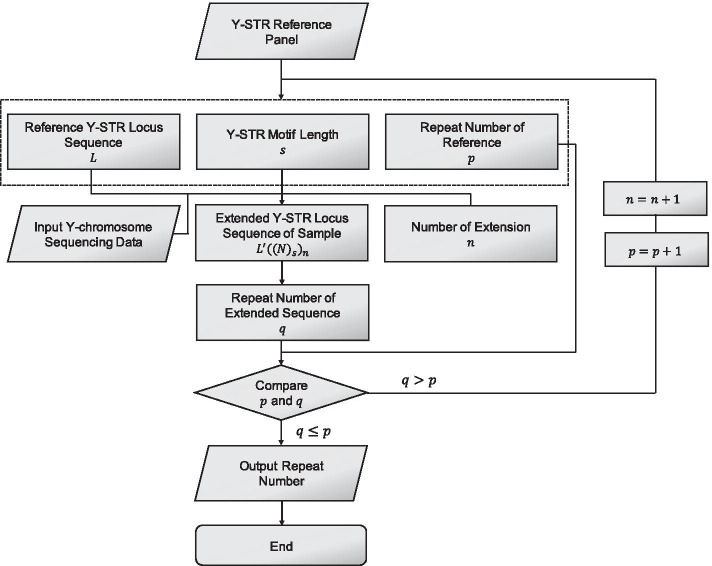
Flow chart of Y-STR genotyping in Y-LineageTracker. The workflow of Y-STR genotyping is implemented in Y-LineageTracker. Based on the Y-STR reference panel, Y-LineageTracker genotypes Y-STRs from Y-chromosome NGS data with high genotyping rate and accuracy

As shown in the flow chart (Fig. 2), to genotype a specific Y-STR locus, Y-LineageTracker firstly gets the reference Y-STR locus from the reference panel. Consider the repeat sequence of the reference Y-STR locus is $L$, which can be represented as: $L = \left( {M_{1} } \right)_{{p_{1} }} \left( {M_{2} } \right)_{{p_{2} }} \cdots \left( {M_{n} } \right)_{{p_{n} }}$, where $M_{1} ,M_{2} \cdots M_{n}$ are motifs of the Y-STR locus, $p_{i}$ represents the repeat number of the ${\text{i}}^{{{\text{th}}}}$ motif. Thus, the sum of repeat number of this Y-STR locus is: $p = \mathop \sum \limits_{i = 1}^{n} p_{i}$.

Based on this sequence information, let the initial sequence $R$ of the Y-STR locus of one sample be forward extended from $L$: $R = L^{\prime}\left( N \right)_{s}$, where $L^{\prime}$ is the sequence at the same position of the reference and $L^{\prime} = \left( {M_{1} } \right)_{{q_{1} }} \left( {M_{2} } \right)_{{q_{2} }} \cdots \left( {M_{n} } \right)_{{q_{n} }}$, $s$ represents the motif length of the Y-STR locus, $\left( N \right)_{s}$ represents the sequence with equal motif length behind this Y-STR locus and $q_{i}$ represents the repeat number of the ${\text{i}}^{{{\text{th}}}}$ motif on initial sequence $R$. As a result, the total repeat number of this extended sequence is: $q = \mathop \sum \limits_{i = 1}^{n} q_{i} .$ Finally, the repeat number of $R$ can be compared with the reference $L$: 1) if the $q$ is equal to or less than $p$, the repeat number is $q$, 2) if the $q$ is equal to $p + 1$, extend the sequence in the same way again and replace $p$ to $p + 1$ and update $q$ of the extended sequence, and repeat this step until the $p$ is equal to updated $q$, which means there are no more motifs extended from the sequence.

## Results and discussion

### Example outputs

**Fig. 3:**
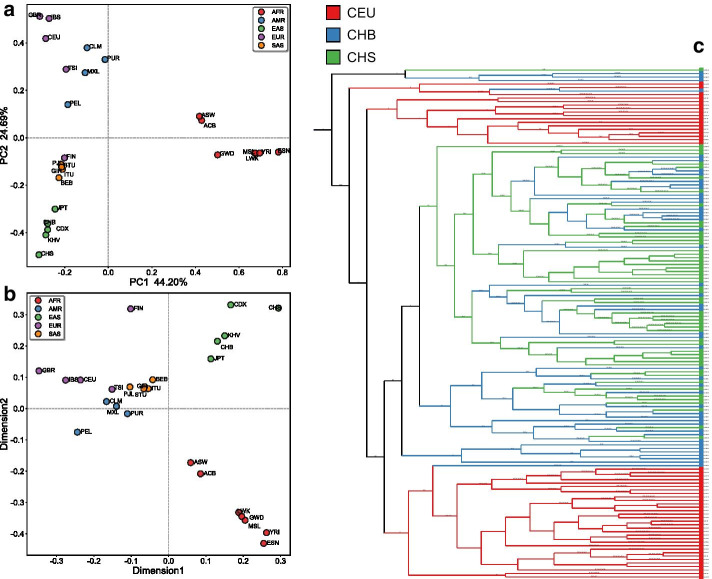
Example output figures of NRY haplogroup analysis performed by Y-LineageTracker. **a** Principal component analysis (PCA) and **b** multidimensional scaling (MDS) performed by Y-LineageTracker respectively, based on NRY haplogroups of 1, 233 male samples from the 1000 Genomes Project data set phase 3. **c** Phylogeny analysis performed by Y-LineageTracker, based on NRY haplogroups of 147 male samples from the 1000 Genomes Project CEU, CHB, and CHS

For NRY haplogroup module, analysis workflow starts from the NRY haplogroup classification. Additional file 3 gives an example of NRY haplogroup classification results of 1,233 male samples from the 1000 Genomes Project [43]. The haplogroup classification results provided by Y-LineageTracker, were compared and validated with the published NRY haplogroup results of the 1000 Genomes Project [1]. We reassigned the terminal mutation makers of the published haplogroup results to the latest ISOGG Y-DNA tree (2019 version) as the haplogroups to be compared with Y-LineageTracker. The results indicated that the NRY haplogroups classified by Y-LineageTracker are consistent with the published results with relatively higher resolution (Additional file 4). In other words, Y-LineageTracker provides sufficient performance to output reliable haplogroup classification results, which is important for subsequent analyses based on NRY haplogroups. Further, based on the haplogroup results, we applied PCA and MDS of clustering analysis function in Y-LineageTracker to directly produce figures that display the population structure of the studied samples (Fig. 3a, b). We also performed phylogenetic analysis in Y-LineageTracker to construct an NRY phylogenetic tree using haplogroup classification results of male samples of Han Chinese in Beijing (CHB), Southern Han Chinese (CHS), and Utah Residents with Northern and Western European ancestry (CEU) from the 1000 Genomes Project (Fig. 3c).

**Fig. 4:**
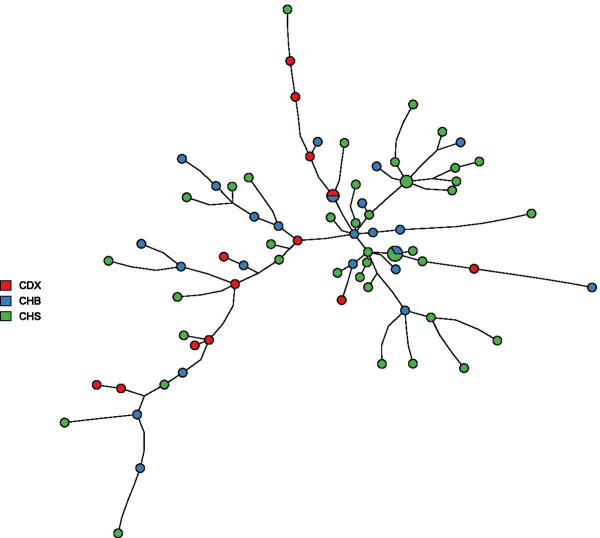
Example output figure of Y-STR network analysis performed by Y-LineageTracker. Network analysis of NRY haplogroup O2 performed by Y-LineageTracker under the minimal Y-STR panel, based on Y-STR haplotypes of 77 male samples from the 1000 Genomes Project CHB, CHS, and CDX

In order to analyze populations of close relationship to a fine-scale, Y-STR analysis within the same NRY haplogroup is recommended. We selected Chinese samples (CHB, CHS, and CDX) with NRY haplogroup O2 and genotyped Y-STRs under the minimal Y-STR genotyping panel. We next applied the network analysis function to do network analysis based on the Y-STR haplotypes (Fig. 4). The result showed that the nodes in the network are admixed, which indicates that there is no significant difference in Y-STR holotypes among three Chinese populations under the haplogroup O2.

### Performance comparison

**Fig. 5:**
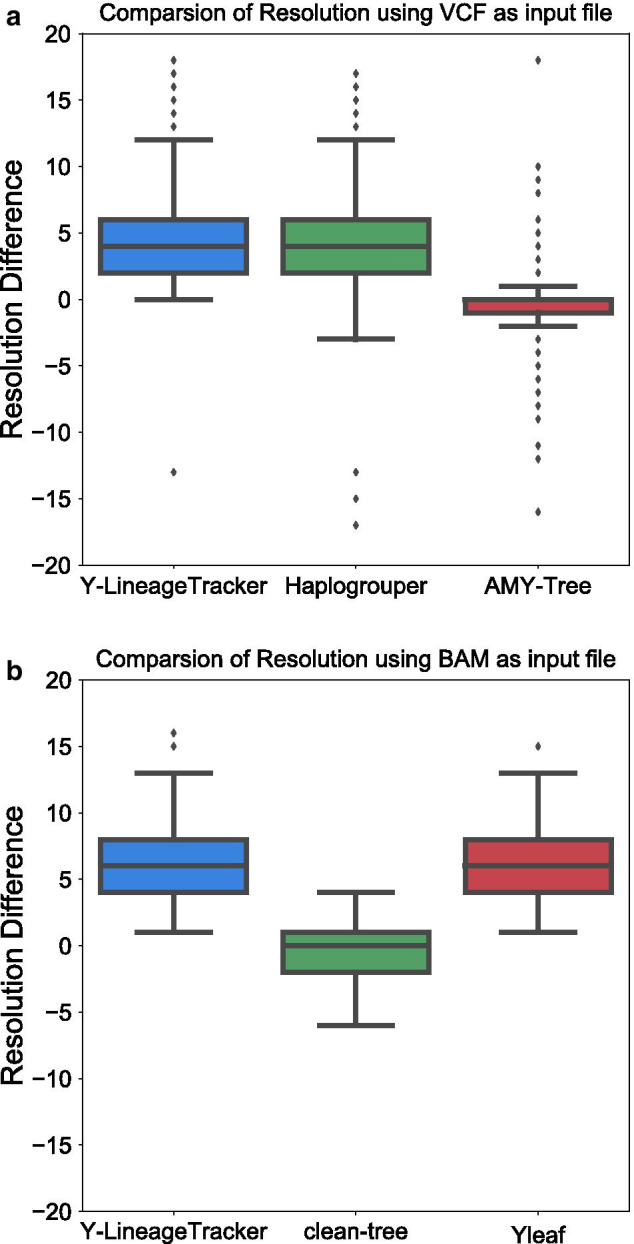
Resolution comparison of NRY haplogroup classification tools. Resolution differences of NRY haplogroup classification tools that use **a** VCF and **b** BAM as input files respectively, compared with published NRY haplogroup results of the 1000 Genomes Project. The difference value is calculated by the resolution of haplogroups classified by tools minus the resolution of the published haplogroups. Positive values indicate the resolution of haplogroups is higher than those that are published, and negative values indicate the resolution of haplogroups is lower than those that are published

**Fig. 6:**
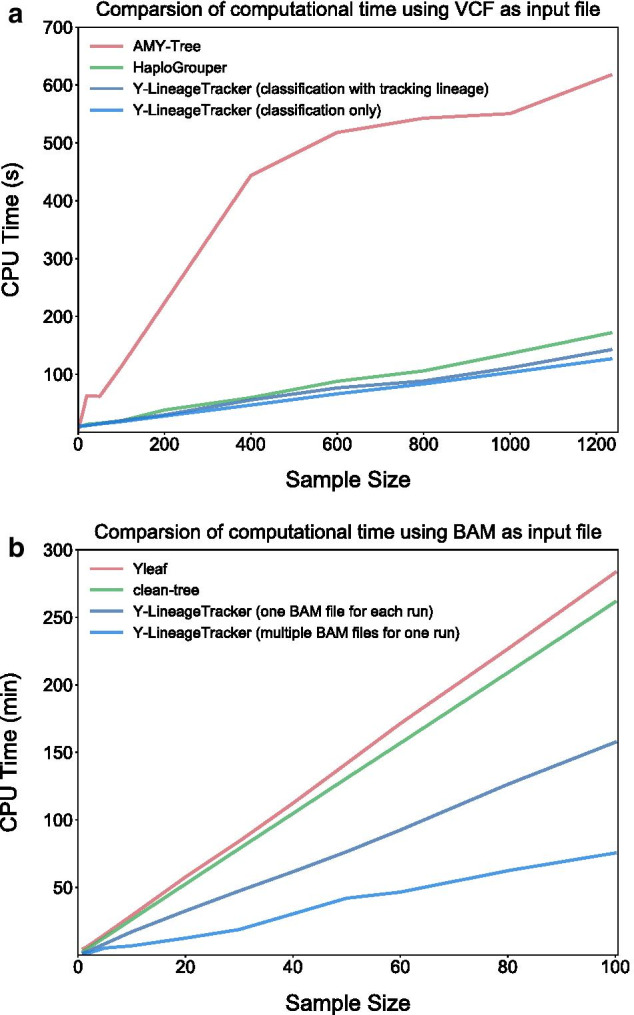
Time costs of NRY haplogroup classification tools. Comparison of time cost of NRY haplogroup classification tools that use **a** VCF and **b** BAM as input files, respectively. The evaluation of time costs is performed on the local machine with 64-bit Red Hat 6.3, CPU AMD Opteron (TM) Processor 6234, and 512 GB RAM. We randomly select male samples with different sample sizes from the 1000 Genomes Project data set phase 3 and perform classification analysis using the same data by different tools. The numbers of variants of VCFs are 62, 043

To evaluate the Y-LineageTracker performance, we compared Y-LineageTracker to other published NRY haplogroup classification tools in different ways. We referred to published NRY haplogroup results of samples from the 1000 Genomes Project [1] and used sequencing data of male samples from the 1000 Genomes Project [43] as input data to run different tools and compare the classification results and time costs. Results showed that Y-LineageTracker performs better with respect to data compatibility for supporting NGS data in VCF or BAM format and can take multiple files as input data to start NRY haplogroup classification analysis (Table 4). The comparison of haplogroup classification results confirmed that (1) when the input file is VCF, Y-LineageTracker classifies NRY haplogroups with higher resolution than the AMY-Tree and with sufficient resolution as good as the HaploGrouper (Fig. 5a and Additional file 4), (2) when input files are BAMs, the resolution of haplogroup classification results is higher than that of the clean-tree and is as high as that of the Yleaf (Fig. 5b and Additional file 5). In addition, the runtime comparison showed that no matter whether the format of input data is BAM or VCF, Y-LineageTracker performed faster in haplogroup classification than other published tools (Fig. 6). Our implementation not only provides high-resolution haplogroup classification results but also reduces computational time.

**Table 4:** General comparison of NRY haplogroup classification tools

Tool	Version	Programming Language	Supported Input Format	Sample Input form	Number of Markers	Supported Reference
Y-LineageTracker	1.3.0	Python	VCF, BAM/CRAM	Multiple	74, 570	hg19, hg38
HaploGrouper	1.0	Python	VCF	Multiple	71, 406	hg19, hg38
AMY-Tree	2.0	Perl	VCF-like	Single	5, 925	hg18, hg19
Yleaf	2.2	Python	FASTQ, BAM/CRAM	Single	65, 459	hg19, hg38
clean-tree	2.0	Python, R	BAM	Single	539	hg19

General comparison of currently available NRY haplogroup classification tools. The sample input form means whether the tool can take multiple files or a single file as input to perform analysis

We also compared Y-STR genotyping results with lobSTR [17] under the Y-STR minimal genotyping panel. The results show that the repeat numbers of Y-STR loci genotyped from Y-LineageTracker are almost consistent with the results of lobSTR, and the Y-STR genotyping rate is higher than that of lobSTR (Additional file 6). Our implementation is not only sufficient to genotype Y-STR loci in specific panels but also provide a higher genotyping rate.

### Application scenarios

We have integrated and improved analysis methods for the NRY with sufficient performance. Our methods provide a commonly used analysis pipeline for the NRY both at Y-SNP and Y-STR levels. We prospect that in the future, with the continuous increase of the public NSG data, Y-LineageTracker can facilitate uncovering population history. For example, combined with analyses of mitochondrial DNA, Y-LineageTracker demonstrated the potential for the detection of sex-biased demography. We also look forward to combining with data of autosome, X-chromosome, and mitochondrial DNA to develop and optimize analysis methods to reconstruct paternal lineages of human genetics and evolution from multiple perspectives.

## Conclusions

We developed Y-LineageTracker, an effective and flexible tool that can standardize analysis workflow for human Y-chromosome sequencing data while performing at a high level. There are two major function modules in Y-LineageTracker, based on NRY haplogroups and Y-STRs, respectively. Y-LineageTracker can not only classify NRY haplogroups and genotype Y-STRs from NGS data with high resolution but also support subsequent analyses for NRY haplogroups and Y-STR haplotypes. We propose that Y-LineageTracker provides an analysis framework to investigate human evolution, population history, and sex-biased demography from Y-chromosome NGS data.

### Availability and requirements

- Project name: Y-LineageTracker
- Project home page: https://www.picb.ac.cn/PGG/resource.php
- Operating system: Platform independent
- Programming language: Python3
- Other requirements: Python packages (numpy, pandas, scipy, scikit-learn, matplotlib, ete3, pysam, and networkx)
- License: MIT
- Any restrictions to use by non-academics: none

## Supplementary Information

**Additional file 1: Table S1**. Y-STR reference panel used for Y-STR genotyping. This table gives a detailed reference panel for Y-STR genotyping applied in Y-LineageTracker.

**Additional file 2:** Text.

**Additional file 3: Table S2.** Example output of NRY classification results generated by Y-LineageTracker. This table gives an example of the Y-LineageTracker output result of 1, 233 male samples from the 1000 Genomes Project data set phase 3.

**Additional file 4: Table S3.** Result comparison of NRY haplogroup classification tools on VCF data. This table gives a comparison of NRY haplogroup results of 1, 233 male samples from the 1000 Genomes Project data set phase 3 in VCF, performed by different NRY haplogroup classification tools that can use VCF data as input files, including Y-LineageTracker, HaploGrouper, and AMY-Tree.

**Additional file 5: Table S4.** Result comparison of NRY haplogroup classification tools on BAM data. This table gives a comparison of NRY haplogroup results of 100 male samples from the 1000 Genomes Project data set phase 3 in BAM format, performed by different NRY haplogroup classification tools that can use BAM data as input files, including Y-LineageTracker, Yleaf, and clean-tree.

**Additional file 6: Table S5.** Result comparison of Y-STR genotyping tools. This table gives a comparison of Y-STR genotypes of 100 male samples from the 1000 Genomes Project data set phase 3 in BAM format, performed by Y-LineageTracker and lobSTR.

## Data Availability

Source code is available at https://www.picb.ac.cn/PGG/resource.php. The 1000 Genomes Project data analyzed in the current study are available at https://www.internationalgenome.org/data. Data generated in this study are included in the additional files.

## Abbreviations

DNA: Deoxyribonucleic acid
NGS: Next-generation sequencing
NRY: Non-recombining region of the Y-chromosome
SNP: Single-nucleotide polymorphism
STR: Short tandem repeat
TMRCA: Time to the most recent common ancestor
PyPI: Python package index
ISOGG: International society of genetic genealogy
PCR: Polymerase chain reaction
VCF: Variant call format
BAM: Binary alignment map
PCA: Principal component analysis
MDS: Multidimensional scaling
AMOVA: Analysis of molecular variance
UPGMA: Unweighted pair group method with arithmetic mean
MCMC: Markov chain Monte Carlo
YHRD: Y-Chromosome STR Haplotype Reference Database
CHB: Han Chinese in Beijing, China
CHS: Southern Han Chinese
CEU: Utah residents with Northern and Western European ancestry
CDX: Chinese Dai in Xishuangbanna, China
