# Supplementary material for: Y-LineageTracker: a high-throughput analysis framework for Y-chromosomal next-generation sequencing data
Source: BMC Bioinformatics. 2021 Mar 9;22:114. doi: 10.1186/s12859-021-04057-z (PMC7941695; doi:10.1186/s12859-021-04057-z)
Supplement: Supplementary file 2 — Additional file 2: Text. [file 12859_2021_4057_MOESM2_ESM.docx]

**Y-LineageTracker: a high-throughput analysis framework for Y-chromosomal next-generation sequencing data (Supplementary Text)**

**NRY haplogroup simplification in clustering analysis**

To perform clustering analysis for NRY haplogroups on a relatively similar resolution, Y-LineageTracker provides a simplification option *--level* in clustering analysis to balance different resolution of haplogroup results.

Consider that there are $n$ NRY haplogroups in a set: $Y=\left\{ {hg}_{1}, {hg}_{2},\cdots{hg}_{n} \right\}$, and the initial resolution of one haplogroup $hg$ is $r$. The argument of simplification option can be *l*, *min* and *auto*. The final resolution of the haplogroup $r^{'}$ is defined as follows:

$$r^{'}=\left\{ \begin{aligned} &l ((1<l\leq max\left( r_{1}, r_{2},\cdots,r_{n} \right)) \\ &min\left( r_{1}, r_{2},\cdots r_{n} \right) \\ &auto({hg}_{1}, {hg}_{2},\cdots{hg}_{n}) \end{aligned} \right.$$

For the *auto* function, the program will:

1.Group the haplogroups into the corresponding Y-DNA tree main trunks.

2.Simplify haplogroups of the main trunk with parsimony approach.

In the second step, consider that there are $m$ haplogroups in the main trunk, and the initial set of this trunk is $M_{1}$:

$$M_{1}:=\{{hg}_{1}, {hg}_{2},\cdots{hg}_{m}\}$$

$M_{1}\in Y$, and the frequency of haplogroups in $M_{1}$ are $f_{1}, f_{2},\cdots f_{m}$. Then, select the haplogroup in $M_{1}$ with the highest frequency as the initial ${hg}_{m}$:

$${hg}_{m}={hg}_{max(f1, f2, \cdots fn)}$$

The resolution of ${hg}_{m}$ is $r_{m}$, and then replace downstream haplogroups of ${hg}_{m}$ with the same as it, and get the $M_{2}$:

$$M_{2}:=\left\{ {hg}^{'} | {hg}^{'}=\left\{ \begin{aligned} &{hg}_{m} (\mathrm{if}hg is the downstream of {hg}_{m}) \\ &hg (\mathrm{if} hg is not the downstream of {hg}_{m}) \end{aligned} \right.,hg\in M_{1} \right\}$$

Next, replace the ${hg}_{m}$ to newer one step by step if its upstream haplogroup exists:

$$M_{3}:=\bigcup_{j=1}^{r_{m}-1} \left\{ {hg'}^{'} | {hg'}^{'}=\left\{ \begin{aligned} &{hg}_{r=j}^{'} \left( {hg}^{'}={hg}_{m} \right) \\ &{hg}^{'} \left( {hg}^{'}\neq{hg}_{m} \right) \end{aligned} \right.,{hg}^{'}\in{M_{2}}_{(r=r_{m}-j)} \right\}$$

Finally, remove the ${hg}_{m}$ and the haplogroups that have been replaced to ${hg}_{m}$, and repeat the second step from $M_{3}$ until $M_{n}= \emptyset$.

3.Get all the ${hg}_{m}$ in each second step as haplogroups to perform clustering analysis.

**Raw output of PAML mcmctree**

The mcmctree program outputs four files: 1) the mcmc file is the raw output of the MCMC and records the MCMC runs, 2) the out file is a summary of the running results, 3) the file “SeedUsed” contains the random seed used to initialize the MCMC, 4) the file “FigTree.tre” gives a posterior tree in nexus format, which can be visualized in FigTree (<http://tree.bio.ed.ac.uk/software/figtree>). Y-LineageTracker summarizes the output results by integrating mcmc file and out file to generate a new output file containing the estimated time of each tree node.

**Platform testing**

Because Python is a cross-platform language, we expect that Y-LineageTracker can run on the most commonly used operating systems. To confirm that Y-LineageTracker can run on common platforms, we tested Y-LineageTracker on Windows (Win7), Linux (Red Hat 6.3) and macOS (Mojave 10.14.6). Y-LineageTracker provides the command *LineageTracker test* to check whether the program is able to run correctly, this command can test most functions implemented in Y-LineageTracker with the toy data provided by itself. As a result, Y-LineageTracker can be successfully installed via PyPI and run testing command correctly on these three platforms. Our testing results support the conclusion that Y-LineageTracker can run on Windows, Linux and macOS.
